# Spatial distribution of unscheduled hospital admissions for chronic obstructive pulmonary disease in the central area of Asturias, Spain

**DOI:** 10.1186/s12890-023-02395-7

**Published:** 2023-03-28

**Authors:** Isabel Martínez-Pérez, Verónica González-Iglesias, Valentín Rodríguez Suárez, Ana Fernández-Somoano

**Affiliations:** 1 Departamento de Medicina, IUOPA-Área de Medicina Preventiva y Salud Pública, Universidad de Oviedo, C/Julián Clavería s/n, Oviedo (Asturias), 33006 Spain; 2 Dirección General de Salud Pública. Consejería de Salud, Principado de Asturias. C/Ciriaco Miguel Vigil, 9, Oviedo, 33006 Spain; 3 CIBER Epidemiología y Salud Pública (CIBERESP) - Instituto de Salud Carlos III, Monforte de Lemos Avenue, 3-5, Madrid, 28029 Spain; 4 Instituto de Investigación Sanitaria del Principado de Asturias (ISPA), Roma Avenue s/n, Oviedo, Asturias, 33001 Spain

**Keywords:** COPD, Chronic obstructive pulmonary disease, Spatial distribution, Disease mapping

## Abstract

**Background:**

Chronic obstructive pulmonary disease (COPD) is one of the major causes of mortality worldwide and also reports high morbidity rates and the global burden COPD has continued to rise over the last several decades. The best-known COPD risk factors are tobacco smoke and air pollution, but genetics, age, sex, and socioeconomic status are additional factors. This study aimed to assess the spatial distribution of unscheduled COPD hospital admissions of men and women in the central area of Asturias during 2016–2018 and identify trends, spatial patterns, or clusters in the area.

**Methods:**

Unscheduled COPD hospital admissions in the central area of Asturias were registered, geocoded, and grouped by census tracts (CTs), age, and sex. Standardized admission ratio, smoothed relative risk, posterior risk probability, and spatial clusters between relative risks throughout the study area were calculated and mapped.

**Results:**

The spatial distribution of COPD hospital admissions differed between men and women. For men, high-risk values were located primarily in the northwestern area of the study, whereas for women the cluster pattern was not as clear and high-risk CTs also reached central and southern areas. In both men and women, the north-northwest area included the majority of CTs with high-risk values.

**Conclusions:**

The present study showed the existence of a spatial distribution pattern of unscheduled COPD hospital admissions in the central area of Asturias that was more pronounced for men than for women. This study could provide a starting point for generating knowledge about COPD epidemiology in Asturias.

## Background

Chronic obstructive pulmonary disease (COPD) is one of the major causes of mortality worldwide. According to the World Health Organization, COPD is the third leading cause of death globally and is a common, preventable, and treatable disease characterized by persistent respiratory symptoms and airflow limitation [1]. Tobacco is the main risk factor associated with COPD. Atmospheric pollution and genetic factors, age, sex, socioeconomic status, or previous diseases such as asthma may further play roles in the disease [2, 3].

Chronic obstructive pulmonary disease is generally perceived as a disease affecting elderly people, especially men, although several studies note that the prevalence of COPD is growing rapidly in women and that the disease affects more younger women than younger men [4]. Sex differences in COPD risk factors, including not only tobacco use but also occupational and non-occupational exposure, genetic factors, or infections, may explain the difference in prevalence between the sexes [5, 6].

Disease mapping analyses deals with the observed counts of health events on sets of areal units [7] and constitutes a series of methods extending small area estimation to directly utilize the spatial setting and assumed positive spatial correlation between observations [8]. Disease mapping helps to elucidate the spatial variation of disease and identify important public health determinants that may be crucial in guiding prevention and control programs [9]. Small-area disease mapping studies have become an established technique in geographic epidemiology. These studies aim to determine the spatial variation in disease risk, quantify the amount of spatial heterogeneity and associated patterns, and highlight areas of elevated or reduced risk [10]. Spatial analysis studies, that take into account a specific territory as area of study, of respiratory health disease and social inequalities have been conducted at differing scales and levels of spatial disaggregation [11–13].

Short-term exposure to air pollution parameters – especially particulate matter (PM)- has been associated with hospital admissions for COPD and asthma [14–17], but also socioeconomic indicators play an important role in COPD hospital admissions and mortality rates [4, 11, 18]. Climate parameters such as temperature or humidity have also shown a relationship with COPD hospital admissions [12, 19].

Long-term exposure to air pollution and living close to high-traffic roads (i.e., being exposed to traffic-related NO_2_) has been associated with the prevalence of COPD in Sweden and Denmark [20, 21], and consecutive cross sectional studies conducted among women in the Rhine-Ruhr Basin of Germany, have pointed out that chronic exposure to PM_10_, NO_2_ and living near a major road increase the risk of developing COPD [22].

In Spain, studies conducted in the last 20 years have identified an increasing prevalence of COPD in the population older than 40 years and report prevalences of 9.1% in 1997, 10.2% in 2007, and 11.8% in 2020. Despite its growing prevalence, COPD has an underdiagnosis rate as high as 74.7% [3]. In addition to the high prevalence of this disease, COPD also refers one of the higher morbidity rates. According to the last health survey carried out in Asturias for the year 2017 [23], a 6.3% of the population relate to suffer from respiratory diseases, being the most important chronic bronchitis, emphysema and COPD, diseases that are been studied here. For men, this percentage is slightly higher, a 6.9%, in comparison with a 5.9% for women. In particular, the National Hospital Morbidity Survey for 2019 shows that Asturias has one of the higher rates of hospital morbidity due to chronic obstructive pulmonary diseases and bronchiectasis, with a rate of 294 hospital discharges per 100,000 inhabitants, significantly higher than the Spain global rate of 220 [24] and just below La Rioja Autonomous Community. Furthermore, respiratory diseases constitute the third leading cause of mortality in Asturias, with an amount of 1,617 deaths over 2015, a 12% of the overall mortality [25]. Additionally, COPD generates high demand for hospital care, especially patients with more severe disease (older, severe bronchial obstruction and hypoxemia) and thus have an effect on significant of hospitals costs [26, 27].

The province of Asturias in Northern Spain has the most aged population in Spain [28] and exhibits considerable asymmetry in the distribution of its population: 70% of the population lives in central Asturias, which has noticeable differences between urban and rural areas. This study aimed to assess the spatial distribution of unscheduled COPD hospital admissions for men and women in the central area of Asturias during 2016–2018 and identify trends, spatial patterns, or clusters in the area.

## Methods

**Fig. 1:**
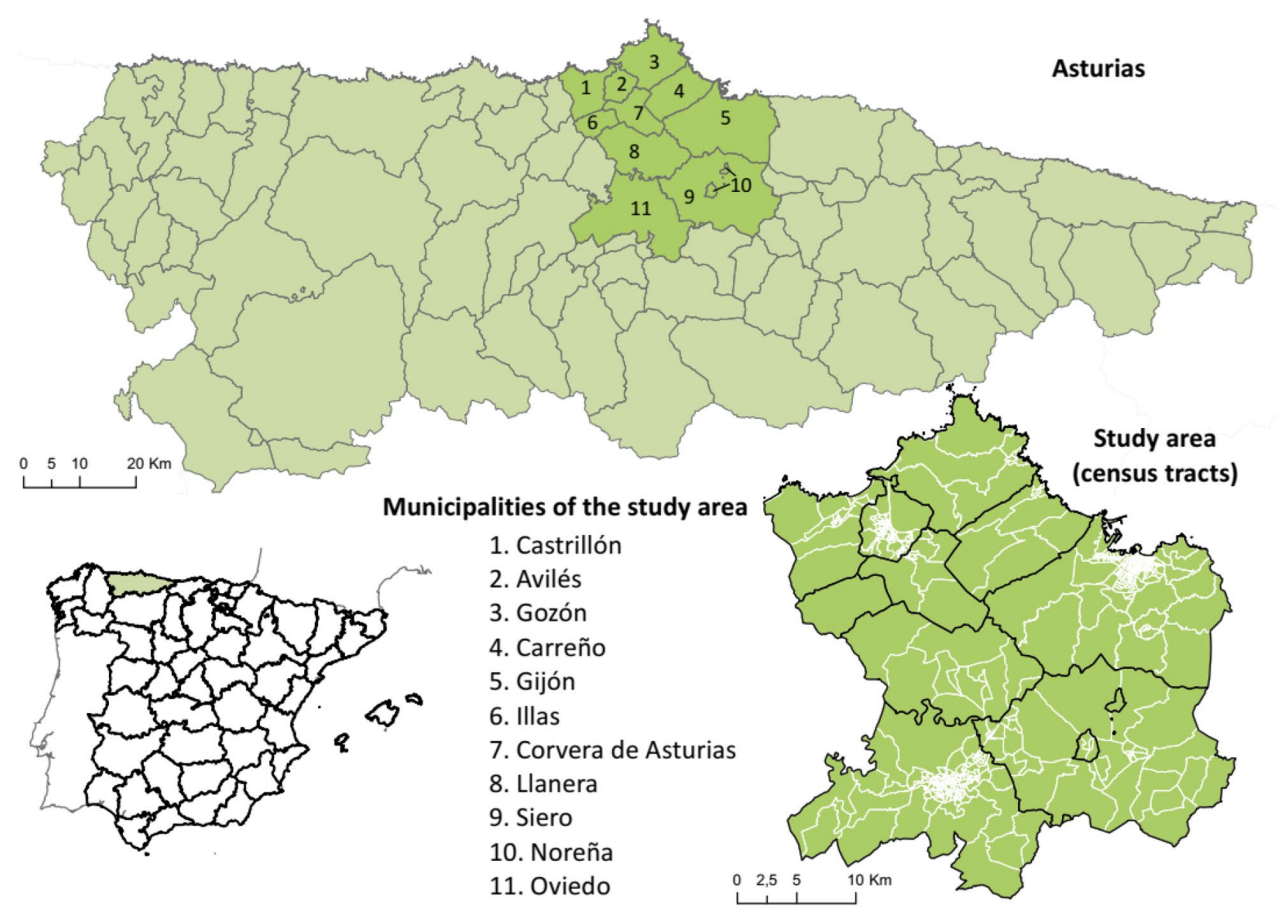
Location of the study area. The study area comprised eleven municipalities in the central area of Asturias, an Autonomous Community of Spain

The study area was the central area of Asturias Province in Northern Spain (Fig. 1). The area has 11 municipalities and a total population of 705,968 inhabitants—roughly 67% of the population of the Asturias region. This area’s four municipalities with the highest populations are Gijón (273,422), Oviedo (220,567), Avilés (80,114), and Siero (51,969); the remaining municipalities have less than 25,000 inhabitants each. Gijón, Avilés and Oviedo are the major urban municipalities of the area, while the rest are mostly suburban or rural areas. The age group of 40–65 is the most populated for both men and women following by the 14–40 group. This territory corresponds with the most populated area in the region that, globally, covers approximately 1,000 km^2^ with a density rate of 705 inhabitants/km^2^. However, there are important differences across the census tracks (CTs), while the rural areas present wide CTs with less population (less than 1,000 inhabitants/km^2^), the CTs of the city’s centers are small but dense (more than 40,000 inhabitants/km^2^).

The study focused on unscheduled COPD hospital admissions coded using International Classification of Diseases (ICD-10), as J41–J44 (J41 – simple and mucopurulent chronic bronchitis; J42 – unspecified chronic bronchitis; J43 – emphysema; and J44–0ther chronic obstructive pulmonary disease) during 2016–2018. Unscheduled hospital admission is defined as any unplanned hospitalization registered in hospitals facilities, without taking in consideration emergency care that does not require hospitalization.

Data on unscheduled hospital admissions into four hospitals in Asturias—San Agustín University Hospital (Avilés), Cabueñes University Hospital (Gijón), Jove Hospital (Gijón), and Central Asturias University Hospital (Oviedo)—were obtained from the Specialised Care Activity Registry RAE-CMBD – Minimum Basic Dataset, which is regulated by legislation [29] and provided by the Health Service of the Principality of Asturias, responsible of its management, which authorized the utilization and analysis of the database according to the Collaboration Agreement SV-PA-19-03.

Each entry corresponded to one admission event, included a personal ID, sex, date of birth, date of admission and discharge, and main diagnosis (ICD 10), and could be linked to a residential address registered in the Population and Health Resources Identification System, which was used for geographical allocation. All entries with J41–J44 ICD 10 codes were included. Information on sex and age group (< 15, 15–39, 40–64, 65–84, and ≥ 85 years) was collected. Entries with any missing variables were excluded.

CTs of municipalities obtained from the National Institute of Statistics were used as unit areas; a total of 558 CTs were included. The CT cartography applied corresponds to the 2016 data year. The total reference population was determined using the CT codes contained in the dataset of the Municipal Register of Inhabitants supplied by the Asturian Society of Economic and Industrial Studies. The CT constitutes the most homogeneous unit of population, with an average of 1,265 inhabitants in each of the 558 CTs included.

### Procedure

Unscheduled hospital admissions data were geocoded using the addresses registered in the dataset. If possible, data were narrowed down to the house level; if this was not possible, data were located on the CT of a given locality. Geocoding was conducted using various levels of cartographic files: primary house-level data for Oviedo, Gijón, and Avilés were provided by the municipalities while data from the CartoCiudad project [30] of the National Geographic Institute and the National Topographic Database BTN100 of the National Geographic Institute covered rural areas that had poor house-level detail in CartoCiudad. ArcGis software (ESRI. ArcGIS Desktop: release 10.5. Redlands, CA, USA: Environmental Systems Research Institute) tools were used to geocode the dataset. Located health events were grouped by CT, sex, and age.

The data were independently analyzed by sex and were standardized by age group. The standardized admission ratio (SAR) was calculated for each CT and it is designed to reflect the number of hospital admissions at each CT relative to the expected hospital admissions so allows to compare the total number of admissions observed for each CT with those expected according to their populations and taking into account their age structure. However, given the variability of the SAR when used in low populated areas, the smoothed relative risk (SRR) was calculated, as it considers the dependency between different units of analysis. SRR was calculated using the Poisson model’s indirect method and the random effects developed by Besag, York and Mollie [31] as smoothing models to account for the spatial adjacency of CTs in the area. The smoothed relative risk was calculated using the integrated nested Laplace approximation (INLA) procedure [32, 33], using BYM [31, 34] as prior and comparing with iCAR [35] and Leroux [36] priors to identify the most accurate model for the area. Simplified Laplace was used as prior for performing Bayesian inference [33, 37]. To determine which of the models fit better, the deviance information criterion (DIC) [38] and the Watanabe–Akaike information criterion (WAIC) [39] were calculated for each model. The SAR and SRR values are expressed in percentages being a value of 100 the reference (neutral) value for risk, values higher than 100 will stand for a risk excess in the given CT, while values lower than 100 mean a lower risk for the population of that spatial unit.

To determine which CTs were high risk, the posterior risk probability (PP; the probability that the smoothed risk is greater than 100, P(SRR > 100)) was calculated. A PP value ≥ 0.8 indicates a statistically significant excess in admissions that is not due to chance. The Stata v14 (Stata Corporation, College Station, TX, USA) and R version 3.6.1 (Foundation for Statistical Computing, Vienna, Austria) programs were used with the INLA library (R-INLA Project, https://www.r-inla.org/) to calculate the SAR, SRR, and PP.

Furthermore, we run a spatial cluster analysis, to identify areas with similar data, using the Moran index [40], which analyzes the spatial autocorrelations between SRRs across the area’s CTs to determine the existence of spatial randomness (i.e., null hypothesis) or its alternative, the existence of spatial autocorrelation.

We calculated local indicators to detect possible spatial autocorrelations in specific subsets of spatial units. In this way, an index was obtained for each spatial unit studied, allowing us to assess the degree of one spatial unit’s dependence on others. To that end, we used the local Moran statistic proposed by Anselin [41], whose interpretation is similar to that of the Moran index: a statistically significant and positive index results in the presence of a cluster of similar values around one spatial unit; in contrast, a statistically significant but negative index causes a cluster of values (i.e., spatial outliers) to appear around the spatial unit. The results of spatial autocorrelation at the local level were presented using the local indicators of spatial association, which used the local Moran indices calculated for all CTs assessed and allowed the geographical determination of (1) spatial groupings that occur when a spatial unit that registers a high/low value of the variable is surrounded by spatial units that also register high/low values of that variable (i.e., high–high or low–low) and (2) spatial outliers that arise when a spatial unit with a high value of the variable is surrounded by spatial units in which the variable registers small values or vice versa, i.e., high–low or low–high.

## Results

In the study period, 3,814 unscheduled COPD hospital admissions—or 98.7% of total events in the database—were analyzed. The remaining 1.3% of events were impossible to georeference because of incomplete data.

The analyzed data included 2,915 men (76.4% of admissions) and 899 women (23.6% of admissions), indicating a clear preponderance of men over women. Although the most represented age group was the 65–84-year group for both men and women (1,889 men and 461 women), this group represented 64.8% of men and only 51.3% of women. Among women, the 40–64-year age group represented 39.4% of admissions.

**Fig. 2:**
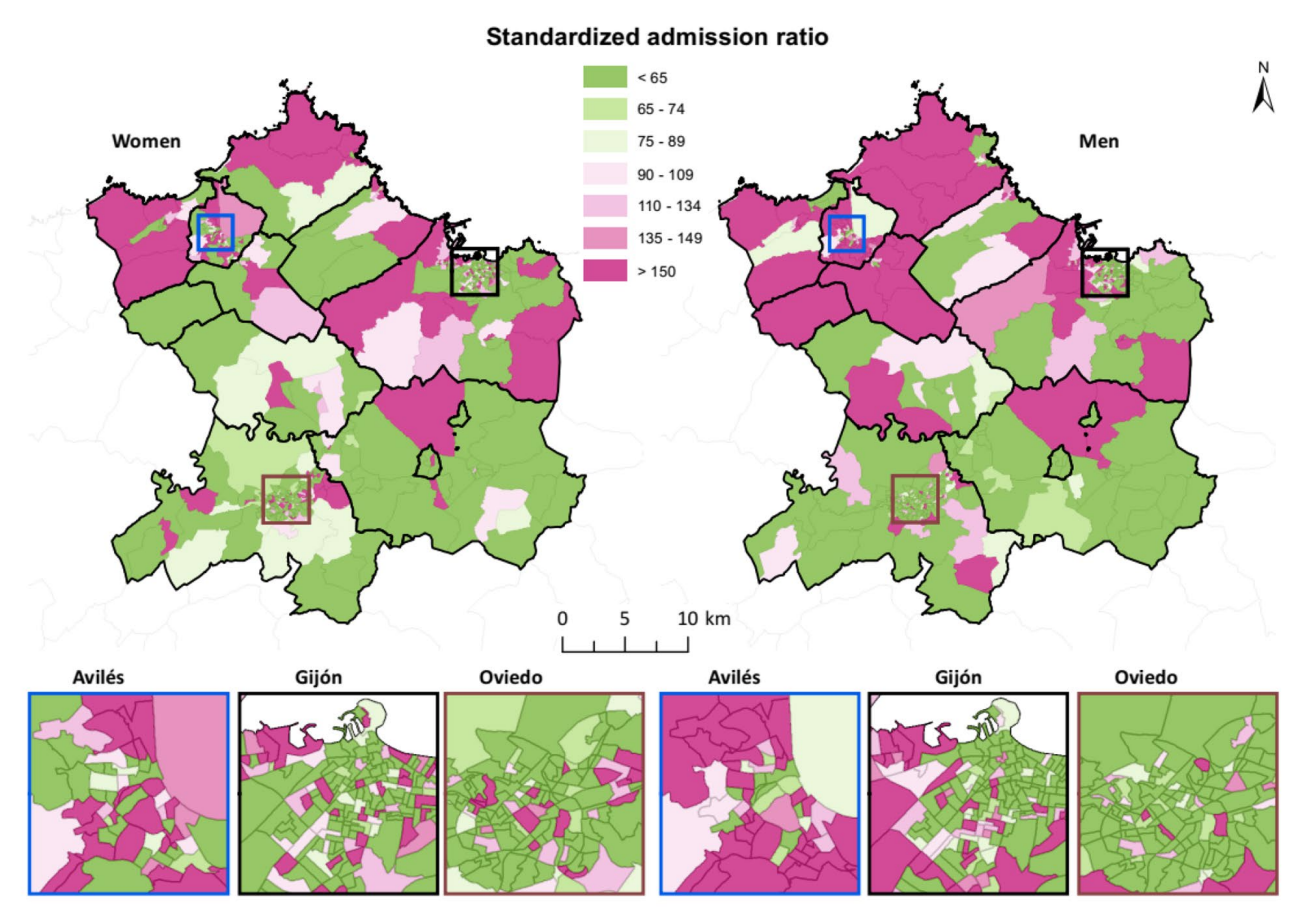
Standardized admission ratio

In both men and women, the representation of SAR had a north-south pattern in which northern CTs showed values over 100 while southern CTs largely had values under 100 (Fig. 2).

**Fig. 3:**
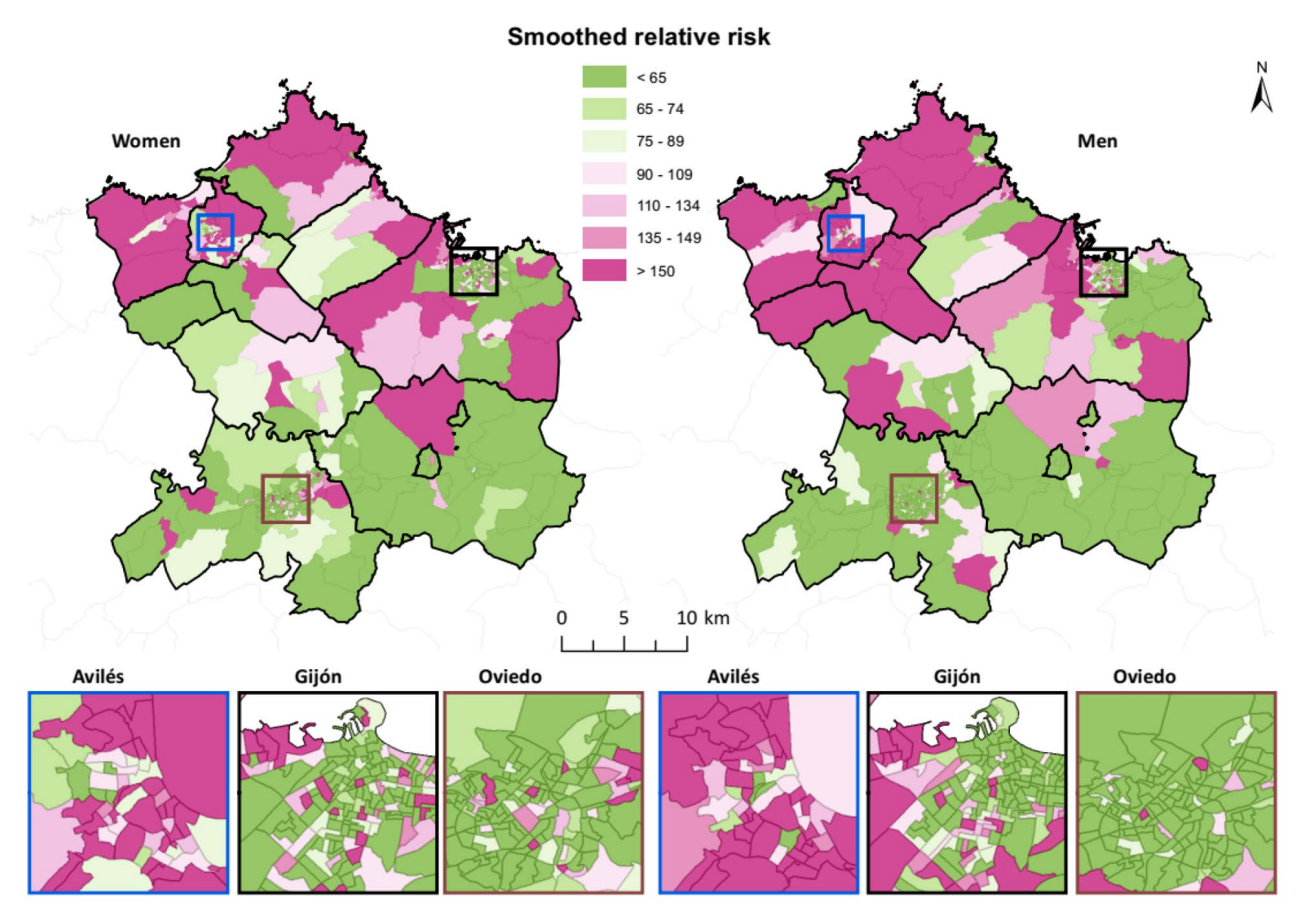
Smoothed relative risk

To allow a better spatial analysis of patterns, the SRR was calculated to smooth SAR values (Fig. 3). In this case, high values were observed in the northern area for both women and men; however, high values for women were distributed in eastern and western areas whereas an extended area of higher values for men was unambiguously observed in the northwest.

**Fig. 4:**
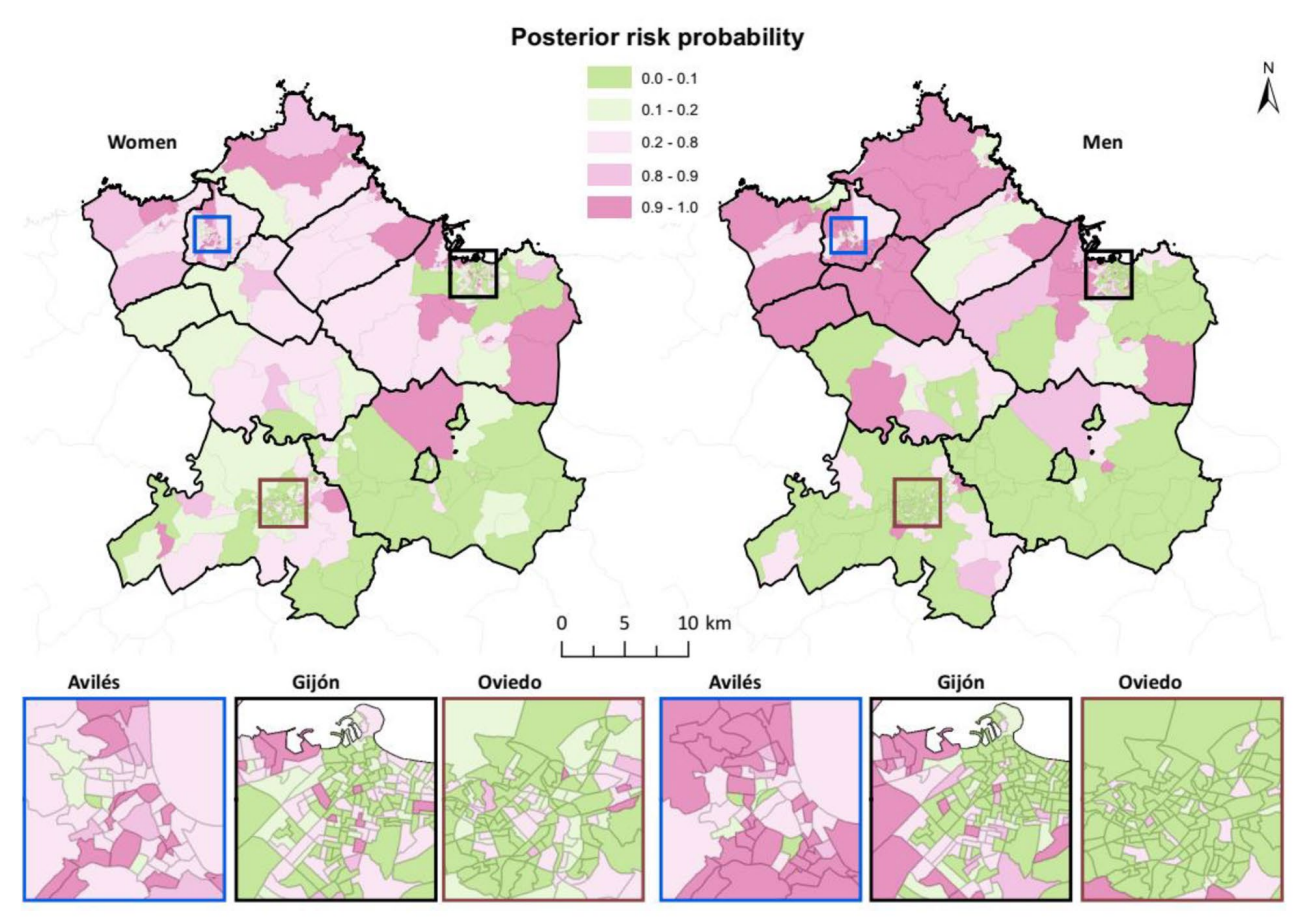
Posterior risk probability

The spatial distribution of the PP (Fig. 4) showed a clear differentiation between women and men. Fewer CTs with values over 0.8 were observed for women than for men and their distribution across the area indicated that the risk of COPD in women was less concentrated in a specific area than in men, for whom high-risk values ​​for COPD hospital admissions were observed in study area’s northwestern CTs.

**Fig. 5:**
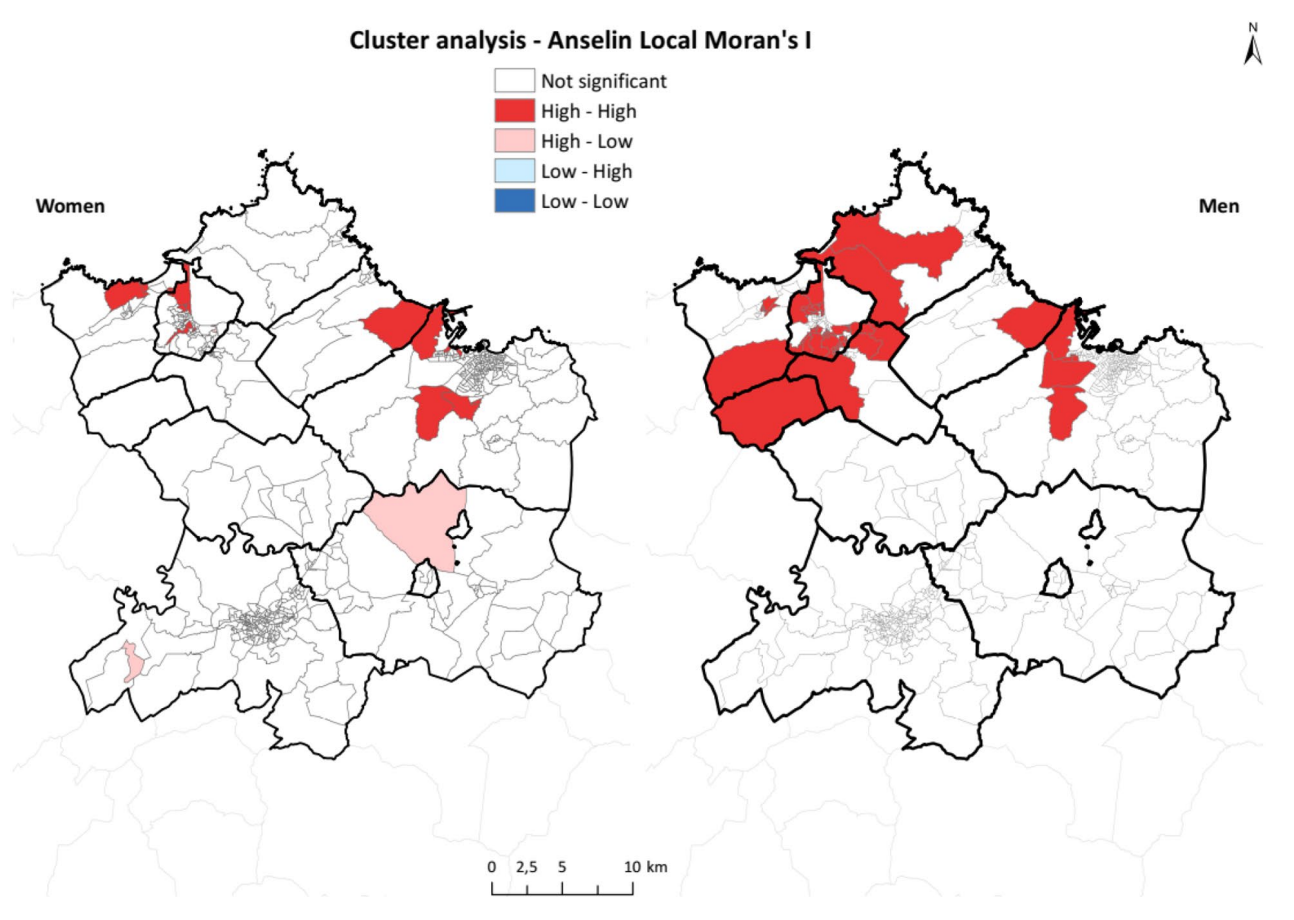
Cluster analysis – Anselin Local Moran index

The cluster analysis (Fig. 5) further showed a more concentrated area for men, with high values located in the northwest and extending to the center until Gijon municipality. For women, the distribution of high values shared between contiguous CTs was limited across the study area, with high-value CTs located in Avilés and Castrillón municipalities, but also in Gijón and Carreño.

## Discussion

The results showed a differing pattern between men and women in the distribution of COPD hospitalizations in the central area of Asturias. The CTs of the northern area registered most of the high values for SRR and PP and the cluster areas were concentrated with high–high values for both men and women. The analysis for men showed a more extensive area of CTs with high-risk values compared with that for women; high-risk values were located primarily in the west but further included CTs in Gijón and Carreño in the central area. The cluster pattern was not as clear for women: CTs with high values were observed in the Avilés and Gijón areas, but PP values above 0.8 were also observed in central and southern areas.

Spatial cluster and spatial aggregation analyses confirmed that, for men, the north-northwest area included most of the CTs with high-risk values, whereas noteworthy patterns were not observed in the rest of the territory. In women, the cluster analysis revealed two areas of high values: one in the northwest area (Avilés and Castrillón municipalities) and the other in Gijón and Carreño municipalities. However, few CTs were involved in both areas. Notably, high-value CTs surrounded by low-value CTs were observed in Siero and Oviedo.

This spatial pattern for SRRs and PPs of COPD hospital admissions in men and women is consistent with findings for unscheduled hospitalizations for ischemic heart disease [42], for which higher rates were observed in northwestern CTs. Air pollution and social inequalities are risk factors shared by COPD and ischemic heart disease share and may drive the prevalence of these illnesses in the area. Various studies [43, 44] have analyzed the association between COPD and ischemic heart disease, a cardiovascular condition that is a major cause of hospitalization in COPD patients [45].

The spatial patterns found in Oviedo, Gijón, and Avilés are similar to those in a study of the geographic variability in COPD mortality in urban areas across Spain [11]. Moreover, notably higher standardized mortality ratios in men were observed in Avilés municipality than in Gijón and Oviedo; this appears to correlate with the spatial distribution of PPs observed in our study.

The results obtained for men defined an area—primarily in the municipalities of Avilés, Castrillón, Corvera, Gozón, and Illas—in which high values were concentrated and the risk of suffering an unscheduled COPD hospitalization was higher than in the rest of the territory. The important industrial activity in this zone of Asturias may be influencing the respiratory health of the population. According to previous studies, air pollution due to industrial activity is related to an increased rate of COPD hospitalization [16] and, together with high-traffic roads exposure, is also linked to COPD prevalence [46]. In the same way, a UK study using UK Biobank data concluded that the prevalence of COPD is associated with higher concentrations of PM_2.5_, PM_10_, and NO_2_ and that the disease has a greater impact on men, individuals from lower-income households, and those in occupations with adverse respiratory exposures [47]. A previous study of air pollution and hospital admissions conducted in the municipalities of Avilés, Oviedo, and Gijón from 2003 to 2018 showed a strong association, more pronounced in men than in women, between hospital admissions for COPD and air pollution mainly caused by NO_2_ [48].

The spatial variability of the distribution of unscheduled COPD hospital admissions in central Asturias suggests the existence of social inequalities that could drive higher COPD prevalence in specific areas compared with others. This finding allows us to generate hypotheses, based on previous knowledge, about risk factors such as air pollution and social inequalities that may influence spatial distribution. Public health measures should be implemented in this high-risk area. Specifically, further studies based on surveillance and associations with possible risk factors are required to elucidate the epidemiology of COPD in the region and guide actions to reduce inequalities.

### Limitations

This epidemiological, ecological and retrospective study has some limitations. The codification process takes place at hospitals and is carried out by specialized personnel according to the guidelines of legal regulations, but thus it is generated by different specialists and hospitals, it is not possible to dismiss some minor error. However, given that this diagnosis is made after discharge and according to all testes made during the hospital stay, even if there were some misclassifications among the data, this should be small and would not affect the result of the study.

Hospitals admissions for COPD are selective in relation to the severity of the disease. In this study we are not analyzing prevalence but we aim to generate hypothesis about the etiology of the illness and the probable factors influencing the inequality distribution found on the territory through the analysis of risk of being admitted to hospital due to COPD. This does not allow us to considerer incidence, because we take into account all hospital admissions across the study period, therefore the same person could have been admitted more than once.

While, the association between air pollution and hospital admissions was observed across the study territory [48]; the poor distribution of air pollution stations makes data estimation inappropriate in the remaining municipalities and could result in exposure misclassification and the impossibility of linking exposure data to CTs.

Finally, the use of CTs is common in epidemiological studies [11, 12] because the CT usually represents the smallest cartographic area with available socioeconomic data. The CTs in this study differ in size because of their urban or rural characteristics; however, given that CTs are relatively homogeneous in population, the negative effects of using such a small area are reduced [49].

## Conclusion

The present study indicates the existence of a spatial distribution pattern of unscheduled COPD hospital admissions in central Asturias that is more pronounced in men than in women. This study could be a starting point for generating knowledge about COPD epidemiology in Asturias. Further studies should be conducted to better identify the underlying causes—including environmental and socioeconomic factors—of the observed spatial variability.

## Data Availability

The datasets used and/or analysed during the current study are not publicly available due to privacy issues given the small size of the units of study, but are available from the corresponding author on reasonable request.

## List of Abbreviations

COPD: Chronic obstructive pulmonary disease
CTs: census tracts
DIC: deviance information criterion
INLA: integrated nested Laplace approximation
PP: posterior risk probability
SAR: standardized admission ratio
SRR: smoothed relative risk
WAIC: Watanabe–Akaike information criterion
